# The Pharmacological Properties of Red Grape Polyphenol Resveratrol: Clinical Trials and Obstacles in Drug Development

**DOI:** 10.3390/nu15204486

**Published:** 2023-10-23

**Authors:** Mohd Farhan, Asim Rizvi

**Affiliations:** 1 Department of Basic Sciences, Preparatory Year Deanship, King Faisal University, Al Ahsa 31982, Saudi Arabia; 2 Department of Biochemistry, Faculty of Life Sciences, Aligarh Muslim University, Aligarh 202002, India; rizvirizviasim@gmail.com

**Keywords:** polyphenol, red grape, resveratrol, physiological effects, pharmacological activity

## Abstract

Resveratrol is a stilbenoid from red grapes that possesses a strong antioxidant activity. Resveratrol has been shown to have anticancer activity, making it a promising drug for the treatment and prevention of numerous cancers. Several in vitro and in vivo investigations have validated resveratrol’s anticancer capabilities, demonstrating its ability to block all steps of carcinogenesis (such as initiation, promotion, and progression). Additionally, resveratrol has been found to have auxiliary pharmacological effects such as anti-inflammatory, cardioprotective, and neuroprotective activity. Despite its pharmacological properties, several obstacles, such as resveratrol’s poor solubility and bioavailability, as well as its adverse effects, continue to be key obstacles to drug development. This review critically evaluates the clinical trials to date and aims to develop a framework to develop resveratrol into a clinically viable drug.

## 1. Introduction

**Figure 1:**
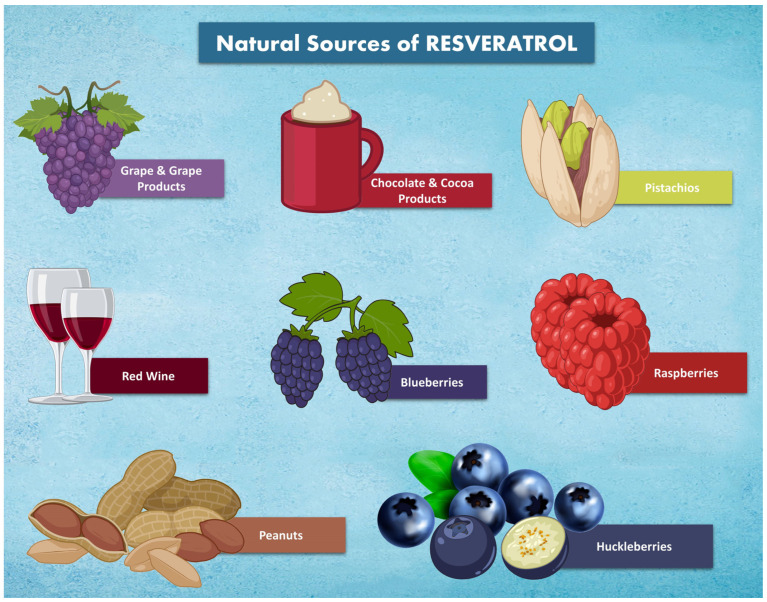
Several resveratrol-containing foodstuffs.

The natural polyphenol resveratrol (*trans*-3,5,4′-trihydroxystilbene) is a stilbenoid. In 1939, Takaoka was the first to successfully extract resveratrol from Veratrum grandiflorum. [1,2]. The skin of red grapes contains the highest concentration of resveratrol. It has also been shown that certain foods, including tea, blueberries, pomegranates, almonds, pistachios, and dark chocolate, contain resveratrol (Figure 1) [3,4].

**Figure 2:**
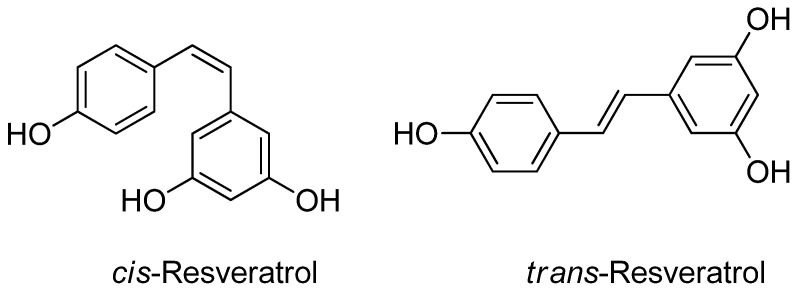
The chemical structure of resveratrol (*cis* and *trans* forms).

Resveratrol comprises a phenol ring connected to a catechol by an ethylene bridge. Two isomeric variants of resveratrol, *cis-* and *trans*-resveratrol, can be distinguished based on their chemical structure (Figure 2). The *trans* form predominates in terms of frequency, and it has been attributed to a variety of biological actions, including the induction of cell cycle arrest, differentiation, apoptosis, and the inhibition of the proliferation of cancer cells [3,4,5].

Resveratrol has been shown to have a broad range of therapeutic effects, such as its anti-inflammatory, antioxidant, platelet-inhibiting, hyperlipidemic, immunomodulatory, anti-carcinogenic, cardioprotective, vasodilatory, and neuroprotective activity [5,6,7,8,9]. It has been reported that resveratrol can sustain or improve human cerebrovascular functions [10], *modulate* in vitro angiogenesis by altering vascular endothelial growth factor (VEGF) expression and the formation of new vascular networks [11], stimulate human immune cell functions [12], boost rat cell viability and proliferation [13], reduce mitochondrial respiratory dysfunction, and boost cellular reprogramming in human fibroblasts derived from patients with a mammalian target of rapamycin (mTOR) pathway deficiency [14]. Resveratrol has been shown to also have neuroprotective [15], hepatoprotective [16], and cardioprotective [17,18] effects. In particular, the polyphenol appears to ameliorate the main risk factors of cardiovascular diseases (CVDs) because it can enhance endothelial function, scavenge reactive oxygen species (ROS), reduce inflammation, inhibit platelet aggregation, and improve the lipid profile, among other things [19,20]. In addition, redox-associated mechanisms were suggested as potential mechanisms through which resveratrol exerts its cardioprotective effects. These redox-associated mechanisms include the maintenance of mitochondrial function during hypoxia/reoxygenation-induced oxidative stress [21], the overexpression of antioxidant enzymes, like peroxidase and superoxide dismutase (SOD) [22], and the regulation of nitric oxide (NO) generation [23].

Resveratrol has been demonstrated to be safe for human consumption in several studies [24,25], although there have also been reports of harmful effects of *resveratrol* in vitro and in vivo [26]. For instance, when resveratrol was given in large quantities, it showed systemic suppression of P450 cytochromes [2]. Several pharmacological interactions involving resveratrol were also discovered and because of the potential for these interactions to reduce the efficacy of the medication, they are considered hazardous [27]. High-dose resveratrol has hormetic effects in vitro (micromolar range in cell culture media) and in vivo (nanomolar range in the blood) [28,29,30], including pro-oxidant effects [29,30,31,32,33,34], but it also has toxic side effects, such as disrupting the thyroid and causing goiter if used for an extended period of time. Thus, it is important to identify the actual biologically effective concentration range at which this compound should be supplemented in human subjects [35,36]. Further studies on pharmacological interactions would allow researchers to address these interactions and understand their cost-to-benefit ratio.

### Resveratrol Bioavailability and Metabolism

**Figure 3:**
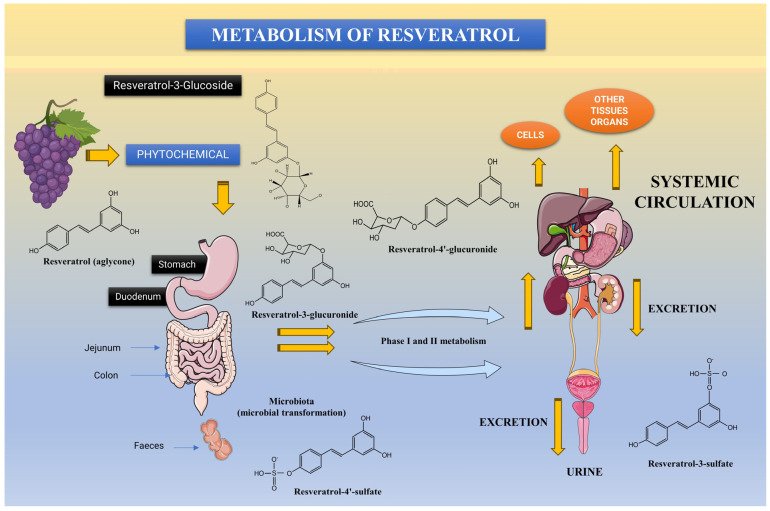
The absorption of resveratrol in the gastrointestinal tract of humans.

There are a number of obstacles in the way of resveratrol being used commercially as a pharmaceutical agent, the most significant of which include resveratrol’s limited bioavailability and quick metabolism. These two factors may reduce resveratrol’s [2,37] effects in vivo. With only a few traces of un-metabolized resveratrol detectable in the plasma after an oral dose of 25 mg [37], it is clear that resveratrol has a very limited bioavailability in the body. Although more than 70% of resveratrol is absorbed in the digestive tract, it is rapidly consumed through three separate metabolic routes after ingestion. According to recent research [38], the bioavailability of resveratrol is determined by its extremely quick sulfate conjugation in the intestine/liver (Figure 3).

The fact that resveratrol is only marginally soluble in water (0.05 mg/mL) hinders its absorption. pH and temperature have significant effects on the stability and solubility of resveratrol [39]. In this context, studies found that the solubility of resveratrol is 64 μg/mL at a pH of 1.2, 61 μg/mL at a pH of 6.8, and 50 μg/mL above a pH of 7.4. Once dissolved in water, resveratrol is only stable at ambient temperature or body temperature under acidic conditions; at higher pH levels, the stilbene is destroyed at an exponential rate. We can infer that resveratrol is most stable in its liquid state when kept at a low pH, cool temperature, and away from oxygen and light [39].

After being ingested, resveratrol moves through the body either through passive diffusion or by forming complexes with transporters such as integrins, albumin, and low-density lipoprotein (LDL) [2,40,41]. Although resveratrol seems to be stable in the stomach’s acidic environment, it may be hydrolyzed to oligomeric phenolics or undergo isomeric conversion. In addition, resveratrol’s glycosylation by resident bacteria in the stomach can result in the absorption-competent stilbenoid glucoside piceid (resveratrol-3-O-beta-glucoside) [2,42]. Intestinal and hepatic conjugation processes also contribute to resveratrol modification. Benzoic, phenylacetic, and propionic acids can be metabolized from resveratrol by intestinal bacteria, whereas phase II metabolism in the liver results in glucuronidated, sulfated, and methylated metabolites that are known to retain some of the biological activity of the parent chemical [2,41,43,44].

The affinity of resveratrol for transport proteins is also connected to its biological effects in vivo. There is a lot of evidence that resveratrol can form complexes with plasma transport proteins, such as human serum albumin (HSA) and lipoproteins, which promote resveratrol stability and activity [45,46,47,48]. To enter various tissues, resveratrol forms complexes with HSA [49,50]. HSA is required in circulation to bind resveratrol, transport it, enhance its uptake by cells, and redistribute it to different cell types [2,48]. In this regard, it has previously been established that epigallocatechin gallate (EGCG), another naturally occurring polyphenolic antioxidant from green tea, can also be bound and stabilized by HSA under aqueous physiological conditions. Consequently, HSA and other plasma proteins may play a pivotal role in mediating the in vivo physiologic effects of resveratrol. Dihydro-resveratrol glucuronides, resveratrol glucuronides, and glucosides are all metabolites of resveratrol, and it is known that resveratrol induces its own metabolism, which raises the activity of phase II hepatic detoxifying enzymes. High levels of these metabolites are detected in human plasma and urine [51,52]. This suggests that free resveratrol may be released locally from these metabolites, as its half-life and plasma concentrations were shown to be 10 times higher than the natural resveratrol component [2,44,53].

## 2. Resveratrol: Pharmacology and Therapeutic Potential

**Figure 4:**
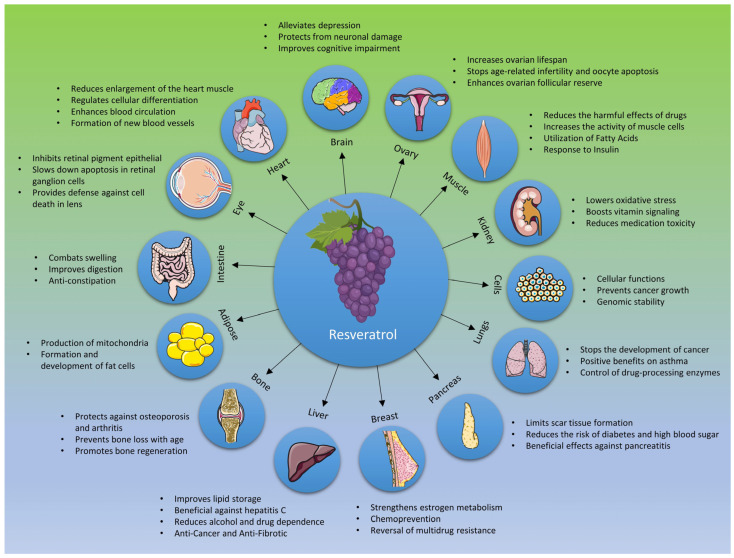
The health benefits of resveratrol consumption in humans.

Some of the most prominent biological actions of resveratrol and its therapeutic potential are summarized in Figure 4 and Table 1. The subsequent sub-sections will elucidate the pharmacological effects associated with moderate consumption of resveratrol with special reference to anti-diabetic effects, cardiovascular effects, neuroprotective functions, and anticancer properties.

### 2.1. Resveratrol in Cardiovascular Health

Heart disease and stroke are the leading causes of death and disability in developed nations [84]. Atherosclerosis is the leading cause of cardiovascular disorders affecting the coronary arteries. Light to moderate alcohol use has been linked to a lower risk of developing type 2 diabetes, increased HDL cholesterol, and decreased lipid oxidative stress. Red wine is superior to other alcoholic beverages in lowering the risk of developing coronary heart disease. It is possible that the synergistic effects of both resveratrol and alcohol come into play in such an action [85].

Resveratrol has been shown in preclinical trials to inhibit LDL oxidation [86]. The effect of red wine on cholesterol is multifaceted, with resveratrol playing a role in hepatic cholesterol and lipoprotein metabolism. The process lowers plasma cholesterol by decreasing cholesterol absorption and its transportation to the liver. Additional effects of resveratrol on cardiovascular disease risk variables include upregulating lipoprotein lipase activity and downregulating low-density lipoprotein circulation [87]. Resveratrol also affects apolipoproteins A and B. In a study, researchers looked at how moderate consumption of red wine, dealcoholized red wine, and gin affected glucose metabolism and lipid profile [88]. Sixty-seven males with a high cardiovascular risk were enrolled in the trial. For four weeks, everyone received 30 g of alcohol each day, which is the same as a standard glass of dealcoholized red wine. A decrease in the Homeostatic Model Assessment for Insulin Resistance (HOMA-IR) and mean-adjusted plasma insulin were observed following wine and dealcoholized wine consumption, while increases in high-density lipoprotein cholesterol, apolipoprotein A-I, and A-II were observed following gin and red wine consumption, and a decrease in lipoprotein was observed following red wine consumption [88]. Paraoxonase 1 is a hydrolytic enzyme that contributes to the protective functions of high-density lipoprotein. A moderate intake of red wine was found to positively alter paraoxonase 1 activity in a healthy Mexican population [89]. A group of researchers [90] looked at the phenomenon of subclinical coronary atherosclerosis. Carotid and femoral artery plaque were measured in this predominantly male sample following polyphenol consumption. Both femoral and carotid subclinical atherosclerosis risk decreased in correlation with increased consumption of flavonoids, while femoral subclinical atherosclerosis risk decreased in correlation with increased consumption of stilbenes. Red wine polyphenols were studied for their potential to counteract age-related declines in vascular function and physical exercise capacity in rats of varying ages (12, 20, and 40 weeks). From week 16 through week 40, rats were treated with either red wine polyphenols or apocynin (an antioxidant and NDPH oxidase inhibitor). Both supplements were found to be effective in reducing endothelial dysfunction, oxidative stress, and abnormal protein expression. Finally, polyphenols in red wine protect against endothelium dysfunction that comes with aging [86]. Seventeen dyslipidaemic postmenopausal women were studied to determine the effects of acute ingestion of red wine and dealcoholized red wine on postprandial lipid and lipoprotein metabolism [86]. Over a six-hour period, acute consumption worsened postprandial lipaemia and increased insulin secretion, but it had no effect on postprandial triglyceride, chylomicrons, or insulin homeostasis. Therefore, it is reasonable to anticipate that long-term use of resveratrol may be good for cardiovascular health [86]. In a particular study, it was shown that moderate consumption of red wine among an elderly population with high cardiovascular risk was associated with a decreased likelihood of metabolic syndrome, abnormal waist circumference, low concentrations of high-density lipoprotein cholesterol, high blood pressure, and hyperglycemia, in comparison to individuals who did not consume red wine [91].

### 2.2. Resveratrol for the Treatment and Prevention of Cancer

Cancer is a leading cause of death all over the world. Each year, it impacts over 6 million people [86]. Chemoprevention is promising for preventing cancer by utilizing either natural or synthetic drugs, or a combination of the two [92]. Resveratrol present in food and drink is thought to be responsible for lowering cancer risk. Stilbenes have been shown to prevent cancer in cell cultures and animals exposed to cancer cells or carcinogenic substances [86]. Colorectal cancer is the third most common kind of cancer, affecting an estimated 1.8 million individuals annually. In most cases, oncogenic mutations accumulate over time in non-cancerous polyps in the intestinal epithelium lining the colon or rectum. If these benign polyps are not caught early enough, they can develop into malignant adenomatous polyps. This development is significantly influenced by environmental factors like nutrition, smoking, alcohol usage, and inactivity. Several studies [93,94] point to the importance of a healthy diet (such as the Mediterranean diet) as a preventative measure against numerous diseases. Cancer prevention is aided by eating foods high in polyphenols and monounsaturated fats, such as those found in the Mediterranean diet [95].

Resveratrol has been investigated for its apoptotic effects on human colon cancer cells (SNU-C4) [96]. Through chromatin condensation and apoptotic body formation, the results demonstrated that resveratrol (100 g/mL) promoted apoptosis in SNU-C4 cells. Resveratrol was found to decrease Bcl-2 expression while increasing Bax and Caspase-3 expression compared to a control group [86]. In order to prevent colon cancer in animals, scientists looked into resveratrol-rich plant extracts like those found in red wine, pomegranate, white grape, and rosemary [97]. Workshop-made cured pork, which is known to promote colon carcinogenesis, had the extracts added to it. Both normal rats and rats provoked by azoxymethane received supplements for a total of 100 days. The number of mucin-depleted foci per colon was found to decrease in response to dry red wine, pomegranate extract, and tocopherol. Incorporating these extracts into cured meat has been proposed as a means of lowering the risk of colorectal cancer associated with eating processed meat [86,97]. It was also determined whether or not red wine extracts were effective in inhibiting the growth of colon cancer cells in vitro and colonic aberrant crypt foci in vivo [98]. Red wine extracts with greater anti-proliferative activity were examined in cells, and the ability to inhibit the development of aberrant crypt foci in mice was found to be the product of a lengthy vinification procedure. Synergistic anti-proliferative effects were also observed between quercetin and *trans*-resveratrol [98].

### 2.3. Resveratrol in Diabetes

The scientific community is becoming increasingly interested in substances that may have anti-diabetic effects. There is hope that such molecules can serve as the foundation for future therapeutic and preventative pharmaceuticals [99]. According to the World Health Organization, almost 500 million individuals will have diabetes mellitus by the year 2025. This condition is part of a more complex metabolic syndrome. Retinal, renal, limb, cardiac, nervous system, and vascular malfunctions, as well as compromised quality of life, and ultimately death, are all associated with this condition [86]. The risk of developing type 2 diabetes is reduced with moderate wine drinking, according to a number of studies [86,100].

In animal studies, simulating type 1 diabetes, a wine concentrate supplemented with natural polyphenols reduced hyperglycemia, brought hemoglobin and erythrocyte counts back to normal, and increased cell survival. Treatment with wine concentrate decreased the activity of catalase and glutathione peroxidase and raised the activity of superoxide dismutase in the plasma of rats with experimental diabetes mellitus [99]. In vitro research [101] looked into the potential anti-diabetic effects of Portuguese red wine. The results demonstrated that both the dealcoholized red wine and the four fractions of red wine produced through solid-phase extraction exhibited potent inhibitory effects against amylase and glucosidase. Monomeric and oligomeric flavan-3-ol molecules are primarily responsible for these actions [86,101]. Researchers examined the effects of co-digesting red wine with models of glucose and whey protein on the digestion, bioavailability, and colonic metabolism of the wine’s polyphenols and constituents. The most significant finding was a decrease in glucose bioaccessibility, which provides more evidence that moderate wine drinking has hypoglycemic effects. Additionally, protein breakdown was slowed, and short-chain fatty acid synthesis (particularly butyric acid) was elevated [102].

### 2.4. Resveratrol in Neuroprotection

**Figure 5:**
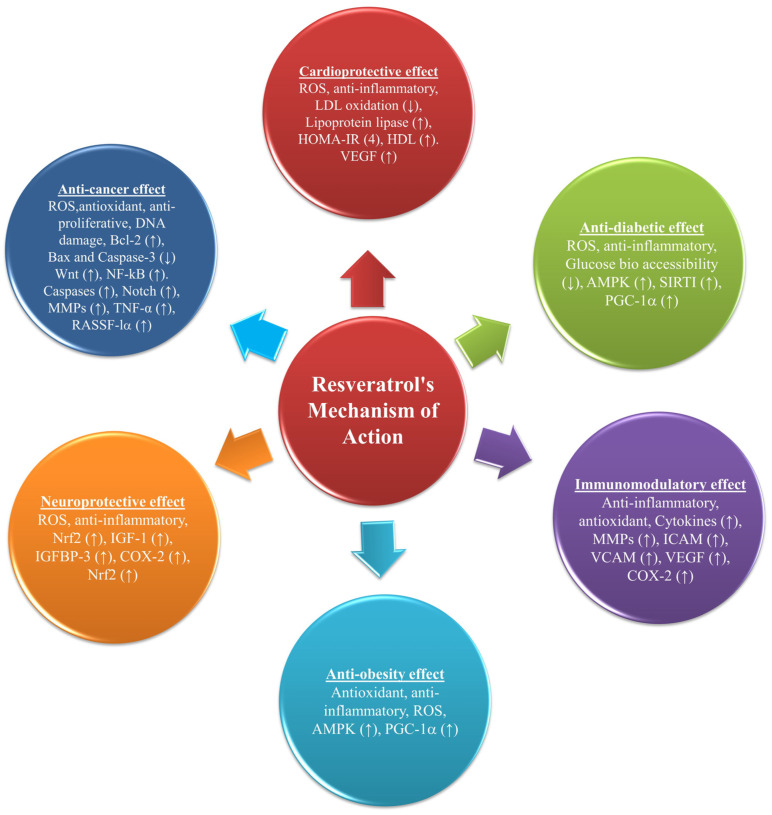
The current knowledge of the action of resveratrol and its prospective therapeutic mechanisms (↑) up and (↓) down; nuclear factor-kB (NF-kB), matrix metalloproteinases (MMPs), 5′-AMP-activated protein kinase (AMPK), intercellular adhesion molecule (ICAM), vascular cell adhesion molecule (VCAM), sirtuin type 1 (SIRT1), tumor necrosis factor α (TNF-α), peroxisome proliferator-activated receptor-γ coactivator 1α (PGC-1α), insulin-like growth factor 1 (IGF-1), insulin-like growth factor-binding protein (IGFBP-3), ras association domain family 1 isoform A (RASSF-1α), pAkt, vascular endothelial growth factor (VEGF), cyclooxygenase 2 (COX-2), nuclear factor erythroid 2-related factor 2 (Nrf2), and Kelch-like ECH-associated protein 1 (Keap1), Homeostatic Model Assessment for Insulin Resistance (HOMA-IR), low-density lipoprotein (LDL), high-density lipoprotein (HDL), wingless-related integration site (Wnt), B-cell lymphoma 2 (Bcl-2), Bcl-2-associated X protein (Bax).

The neuroprotective effects of resveratrol have been the subject of multiple investigations. Pretreatment with resveratrol protected neural stem cells from oxygen–glucose deprivation and activated nuclear factor erythroid 2-related factor 2 (Nrf2) [103]. Piceatoannol, a resveratrol metabolite, prevented glutamate-induced cell death in HT22 neuronal cells [104]. When resveratrol was given to rats, the pre-induction of cerebral ischemia led to the rats’ oxidation indicators dropping, and their superoxide dismutase activity was restored [105]. Glutathione peroxidase and glutathione reductase are necessary for maintaining glutathione in a reduced state. Drinking red wine boosted the enzymes of glutathione metabolism [106]. Red wine powder (freeze-dried with maltodextrin and gum arabic) [107] protects human neuroblastoma SH-SY5Y cell viability when treated with 6-hydroxydopamine. Indeed, red wine powder at a concentration of 150 ng GAE/mL ensured that 88.3% of cells would survive after being exposed to 6-hydroxydopamine cytotoxicity. Polyphenols with numerous hydroxyl groups are effective at preventing the production of mono- and di-adducts that contribute to the formation of advanced glycation end products. This is an effective means of protecting against neurodegenerative disorders [108]. Some of the possible pathways for resveratrol action in different disorders are summarized in Figure 5.

## 3. Human Clinical Trials of Resveratrol

Resveratrol shows promise as a compound that can help cells keep its metabolic balance. Researchers conducted numerous clinical investigations to verify the therapeutic effects of resveratrol on vascular metabolic illnesses in order to better understand its clinical transformative value. This resulted in 244 finished clinical trials and 27 ongoing trials by the end of 2019 [109]. Numerous diseases and disorders, such as diabetes, obesity, cancer, neurological, and cardiovascular diseases, have been the focus of clinical trials investigating the preventive and therapeutic effects of resveratrol. Preclinical and clinical studies have shown that resveratrol can modulate a wide variety of signaling molecules, including wingless-related integration site (Wnt), nuclear factor-kB (NF-kB), cytokines, caspases, Notch, matrix metalloproteinases (MMPs), 5′-AMP-activated protein kinase (AMPK), intercellular adhesion molecule (ICAM), vascular cell adhesion molecule (VCAM), sirtuin type 1 (SIRT1), tumor necrosis factor α (TNF-α), peroxisome proliferator-activated receptor-γ coactivator 1α (PGC-1α), insulin-like growth factor 1 (IGF-1), insulin-like growth factor-binding protein (IGFBP-3), ras association domain family 1 isoform A (RASSF-1α), pAkt, vascular endothelial growth factor (VEGF), cyclooxygenase 2 (COX-2), nuclear factor erythroid 2-related factor 2 (Nrf2), and Kelch-like ECH-associated protein 1 (Keap1) [109,110]. The ability of resveratrol to interact with numerous targets, such as kinases, receptors, and signaling molecules, is the likely explanation for its pleiotropic behavior [109,110]. The major resveratrol clinical trials are listed below in Table 2.

## 4. Resveratrol as an Adjuvant

Contradictory findings in in vivo investigations on resveratrol have been reported, and this discrepancy has been linked to the drug’s low bioavailability, On the other hand, supplementary treatment can help with resveratrol’s subpar bioavailability. Therapeutic benefits can be increased by the synergistic interaction of resveratrol and other bioactive components and micronutrients [137]. This is likely due to an increase in resveratrol bioavailability and a broadening of the metabolic effects of the combined agents. Polyphenols are able to link and interact with other compounds using their hydroxyl groups, which allows them to control the efficacy of other chemicals, including proteins and nutrients [2,138]. Compared to free polyphenols, polyphenol complexes might be more bioavailable, soluble, and absorbable in the small intestine due to their stabilized chemical structure [139]. A potential benefit of combining resveratrol with other treatment modalities is that polyphenol complexes can target numerous metabolic pathways. It is true that resveratrol combined with various therapeutic modalities has been shown to have favorable benefits in a variety of diseases and conditions, including cancer [138].

It has been suggested that the combination of vitamins with polyphenols not only results in synergistic biological effects but also stabilizes, maintains, and supports the action of polyphenols. Combining resveratrol with vitamin D3 has been shown to increase resveratrol’s estrogenic activity and modify ER-mediated transcription [137]. In diabetic nephropathy, resveratrol, and vitamin D3 were found to have synergistic benefits. Combining resveratrol with vitamin D3 has been proven to suppress TNF-α and IL-6 expression more than either medicine alone [140]. The combination of glucan, vitamin C, and resveratrol exhibited a higher suppression of breast and lung tumor growth in in vivo models compared to the individual drugs [138,141].

To combat human papillomavirus (HPV)-positive head and neck squamous cell carcinoma, researchers examined the efficacy of a tri-combination (TriCurin) of three polyphenols (curcumin derived from spice turmeric, resveratrol, and epicatechin gallate from green tea). TriCurin inhibited tumor growth by 85% when administered intratumorally in vivo, and it lowered cell viability, clonogenic survival, and tumor sphere formation in vitro while greatly increasing apoptosis [142]. TriCurin also increased p53 protein levels and decreased HPV16 E6 and E7 [142,143]. In a separate investigation, resveratrol and epicatechin gallate were found to trigger apoptosis in prostate cancer cells at dosages as low as 100 microM [144]. CruciferexTM, a substance derived from cruciferous vegetables, was used in a study of human head and neck squamous carcinoma. CruciferexTM contains a mixture of various polyphenols, including resveratrol. Matrix metalloproteinase (MMP) secretion and cell proliferation were both greatly slowed by the polyphenol mixture [145].

Given the prevalence of cancer and other disorders that require the targeting of several molecular pathways simultaneously, the findings of this study indicate that the combination of polyphenols such as resveratrol, nutrients, and other treatments with additive and/or complementary effects may offer a promising approach to achieve synergistic actions.

## 5. Resveratrol Based Nanoformulations

Various drug carriers have been tried and are being used to improve the poor bioavailability and stability of resveratrol, resulting in a reduced requirement to consume large resveratrol doses and fewer unwanted effects. These can take the form of emulsions, nanoparticles, or liposomes [146,147]. Lipophilic pharmaceuticals can be better stabilized and bioavailable, water-soluble, safe, biodistributed, and biocompatible when encapsulated in solid lipid nanoparticles [148]. The oral bioavailability of resveratrol was improved by up to 335.7 percent when it was loaded into poly-lactic-co-glycolic acid (PLGA) nanoparticles before being administered to rats [149]. The therapeutic potential and efficacy of resveratrol were further increased by nanoparticle formulations, in particular its in vivo anticancer activity in a variety of cancer types. Tumor size was reduced in studies of gliomas, ovarian cancer, and colorectal cancer when resveratrol was administered [150,151]. High-loading resveratrol-loaded gelatin nanoparticles were found to induce cell death via changes in p53, p21, caspase-3, Bax, Bcl-2, and NF-kB expression when utilized in a coculture setting [152]. Using a rat embryonic cardiomyocyte (H9C2) model, researchers discovered that Curcumin-Resveratrol-mP127 (co-loaded curcumin and resveratrol at a molar ratio of 5:1 in Pluronic^®^ F127 micelles) proved cardioprotective by inhibiting apoptosis and reactive oxygen species (ROS) [153]. In addition to cancer treatment, resveratrol has shown therapeutic effects in treating various diseases, therefore further improvements in resveratrol carrier delivery should help mitigate the negative effects of large dosages of resveratrol.

Children with chronic liver illness have stunted growth and development because of poor nutrient absorption [154]. An increase in morbidity and mortality is related to malnutrition, which is an unfavorable prognostic factor in liver transplantation [155]. Current vitamin E supplementation guidelines recommend giving children D-α-tocopheryl-polyethylene-glycol-succinate (TPGS) orally to increase their chances of survival and overall health, although TPGS alone does not prevent spinocerebellar degeneration or lipid peroxidation [156,157]. Micelles loaded with resveratrol have shown a protective action in the liver, boosting the efficacy of TPGS [158]. Through a phase-solubility analysis, the researchers determined that TPGS was suitable for encapsulating resveratrol in micelles; next, resveratrol TPGS formulations were made through solvent casting and solvent diffusion evaporation. Low polydispersity, a somewhat neutral Zeta potential, and small mean diameters (12 nm) were all characteristics of resveratrol TPGS colloidal dispersions [159]. Infrared spectroscopy and differential scanning calorimetry both validated the formulations’ strong drug loading capacity and stable drug release. Resveratrol TPGSs showed reduced toxicity on HaCaT cells compared to empty TPGSs while maintaining the same level of antioxidant activity as pure resveratrol as measured by the DPPH assay. The antioxidant activity of resveratrol and the reduced surfactant toxicity on normal cells suggest that resveratrol TPGS micelles may be able to overcome the obstacles of conventional liver disease therapy [159,160]. Table 3 details the encouraging results obtained from a variety of nanoformulation methods for increasing resveratrol’s biological activity.

## 6. Toxicity and Adverse Effects of Resveratrol

Resveratrol is known for its antioxidant and chemopreventive properties. However, some investigations have shown that it may act as a pro-oxidizing agent [4,29,30], which may paradoxically affect disease pathogenesis. Resveratrol’s antioxidant activity is a consequence of ROS scavenging [174,175] and antioxidant defense upregulation [176]. Resveratrol may modulate gene and protein expression through redox-sensitive intracellular pathways in tissues and cells. Thus, gene expression modifications and increased antioxidant defense system action lead to cell survival and adaptability in oxidative environments [4,177,178]. Resveratrol can also be auto-oxidized to semiquinones and the comparatively stable 4′-phenoxyl radical, producing ROS under certain enzymatic conditions [179]. pH and hydroxyl anions or organic bases affect polyphenol oxidative processes [180].

A study examined the dose–time dependency of acute resveratrol injection on lipoperoxidation levels in male rats’ hearts, livers, and kidneys synchronized with a 12 h dark–light cycle. Resveratrol was an antioxidant in the dark and a pro-oxidant in the light, possibly reflecting the changing ratio of pro- and antioxidant activities in various organs during a 24 h cycle or postprandial oxidative burst [181]. Dietary polyphenols, including resveratrol, have impressive antioxidant and cytotoxic properties. Since every antioxidant is a redox agent, it can become a pro-oxidant, causing lipid peroxidation and DNA damage under certain conditions. Thus, pro-oxidant action may contribute to resveratrol’s anticancer and apoptotic activities [182]. Resveratrol’s pro-oxidant action can damage DNA and stop the cell cycle [178].

Resveratrol can influence many pathways simultaneously, resulting in diverse or even opposite biological effects depending on concentration or treatment period. Although a dose-dependent resveratrol pro-oxidative action causes oxidative stress in cells over short periods of time, less cytotoxicity was identified at the same dose but with longer exposure times. This suggests that surviving cells were more resistant to resveratrol-induced damage, which decreased over time [4,183]. Low resveratrol doses (0.1–1.0 μg/mL) increase cell proliferation, but higher doses (10.0–100.0 μg/mL) cause apoptosis and reduce mitotic activity in human tumors and endothelial cells [184]. Studies have shown that resveratrol has a dual effect on HT-29 colon cancer cells, with low concentrations (1 and 10 μmol/L) increasing cell number and higher doses (50 or 100 μmol/L) decreasing cell number and increasing apoptotic or necrotic cell percentage [185].

Resveratrol is interesting for drug research because it does not have any harmful or debilitating side effects. Resveratrol dosages have been varied in in vivo and in vitro experiments. However, the best dose and route must be determined according to the patient’s needs. In addition, resveratrol causes cell death in tumor tissues but not in normal tissues [182]. The tumor-specific absorption of resveratrol is due to variations in cellular targets and gene expression in cancer cells. It has been shown [186] that lower resveratrol levels may be beneficial, but higher amounts kill tumor cells by pro-apoptotic signaling.

It has also been shown that resveratrol causes cell death in tumor tissues while having little to no effect on healthy neighboring tissues [187]. Because of variations in accessible cellular targets and gene expression, resveratrol is tumor-specific in that its absorption by normal cells is significantly lower than cancer cells. It has been hypothesized that modest dosages of resveratrol may have health benefits, whereas high amounts destroy tumor cells through pro-apoptotic actions [186].

The short-term use (1.0 g) of resveratrol appears to be safe. However, patients with nonalcoholic fatty liver disease may have nausea, vomiting, diarrhea, and liver impairment at dosages of 2.5 g or more per day [188]. Curiously, in long-term clinical trials [189], no serious adverse effects were reported. In fact, a single 5 g dose of resveratrol or a part of that dose spread out over numerous days has been shown to be safe and well-tolerated [190]. It is important to note, though, that these findings may be replicable among sick individuals because the research was conducted on healthy populations. Orally administered resveratrol is metabolized by gut microbiota [191], making it difficult to determine which effects are solely due to resveratrol or both resveratrol and its metabolites, further complicating our understanding of resveratrol dose-dependency and administration route [191].

High doses of resveratrol have been shown to inhibit cell growth and trigger apoptosis in normal cells, confirming the compound’s biphasic actions throughout a wide range of concentrations [192]. Rapid activation of mitogen-activated protein kinase (MAPK) by resveratrol is dependent on MEK-1, Src, matrix metalloproteinase, and the epidermal growth factor receptor. Nanomolar doses (i.e., magnitude less than that required for ER genomic activity) and concentrations possibly/transiently obtained in serum after oral red wine ingestion [193] activate MAPK and endothelial nitric-oxide synthase (eNOS). Mice as young as one years old benefit from resveratrol’s anti-aging properties when consumed in low dosages. Mice fed a dosage of 1800 mg/kg of resveratrol died after three to four months [194]. Despite the common occurrence of diarrhea, studies on the steady-state pharmacokinetics and tolerability of 2000 mg *trans*-resveratrol indicated that it was well-tolerated by healthy subjects [195]. This dosage was given twice a day with meals, quercetin, and alcohol.

Studies highlighting the health advantages of resveratrol all point to the importance of dose and age in eliciting such benefits. Another study that looked at the effects of resveratrol on insulin resistance caused by both aging and re-nutrition found that it increased insulin sensitivity in elderly mice fed a standard diet but had no effect on the insulin resistance status of elderly mice fed a high-protein diet [4]. On the other hand, resveratrol was harmful, lowering aortic distensibility and boosting inflammation and superoxide generation. These results suggest that resveratrol is helpful in a malnourished state of physiological aging, but that it may increase atherogenesis-associated risk factors when combined with high-protein diets in elderly mice, possibly by triggering vascular alterations that are themselves a risk factor for the cardiovascular system [196], which remains to be proven without reasonable doubt.

The biological effects of resveratrol are strongly linked to a hormetic effect (as discussed in the introduction), with low doses generally having beneficial effects and high doses having toxic effects. This biphasic effect on the cellular redox state, which is an antioxidant at low doses and a pro-oxidant at high doses, is believed to be responsible for resveratrol’s hormetic property [2,4]. However, studies on resveratrol have mainly focused on short-term outcomes, leading to controversy [4]. The primary focus should be on resveratrol dosage and interaction with the environment’s redox state, especially when precise redox modulation is needed for physiological function or to prevent harmful effects. More extensive studies in complex models are needed to validate current findings. Despite numerous human and animal studies supporting resveratrol’s beneficial properties, there are not enough clinical studies reporting resveratrol’s harmful effects, and the molecular mechanism of resveratrol’s action needs to be better identified.

## 7. Conclusions and Future Perspectives

This article provides a summary of the research on the health benefits and mechanism of action behind red grape polyphenol resveratrol, including its effects on cardiovascular disease, cancer prevention and therapy, neuroprotection, and diabetes. Studies on both animals and humans show that resveratrol, when consumed in moderation, can have positive health effects. But in order to make resveratrol more promising pharmaceutically, adjustments must be made to its structure and bioavailability. The potential of resveratrol in the treatment and prevention of various diseases warrants further investigation. Additionally, resveratrol’s biochemical mechanism of action has to be thoroughly elucidated. Most importantly, more standardized clinical trial designs are needed to adequately examine the benefits of resveratrol and establish its mechanisms of therapy and prevention of disease.

## Figures and Tables

**Table 1:** A summary of resveratrol’s various pharmacological properties.

Pharmacology	Reference
Anticancer	[54,55,56]
Analgesic and Anti-inflammatory	[57,58,59]
Anti-diabetic	[60,61,62]
Neuroprotection	[63,64,65]
Antiviral	[66,67,68]
Anti-obesity	[69,70,71]
Cardioprotection	[72,73,74]
Antioxidant	[75,76,77]
Anti-aging	[78,79,80]
Nephroprotection	[81,82,83]

**Table 2:** A review of the clinical trials on resveratrol’s potential as a therapeutic molecule.

Clinical Condition	Cohort Size (Numbers)	Resveratrol Dose and Duration	Principal Outcomes of Resveratrol Treatment	Reference
Atherosclerosis	Individuals diagnosed with nonalcoholic fatty liver disease were randomly assigned to either a placebo (*n* = 25) or resveratrol (*n* = 25) group	600 mg/day, 84 days	Plasma ox-LDL, LDL-C/HDL-C, and LDL-C/ox-LDL levels showed no changes	[111]
	Individuals in good health were randomly assigned to either a resveratrol (*n* = 24) or a calorie restriction (*n* = 24) group	500 mg/day, 30 days	A rise in plasma TC and non-HDL cholesterol but no change in plasma TG, HDL-C, LDL-C, or apolipoprotein A1	[112]
	Randomized groups of patients with carotid stenosis >70% and a request for surgical intervention were given either Cardioaspirin^®^ and Aterofisiol^®^ (*n* = 107) or Cardioaspirin^®^ and placebo (*n* = 107)	20 mg/day, 25 days	Decreased dry weight of lipid and cholesterol in removed plaques (0.232 ± 0.018 vs. 0.356 ± 0.022; 0.036 ± 0.006 vs. 0.053 ± 0.007 mg/mg dry weight, respectively)	[113]
	Randomized placebo (*n* = 28) and resveratrol (*n* = 28) groups of patients with type 2 diabetes mellitus and coronary heart disease	500 mg/day, 30 days	No change in plasma TG, TC, or LDL-C; HDL-C plasma levels increased; TC/HDL-C plasma levels dropped	[114]
	Stable coronary artery disease patients (*n* = 10) were given placebo or resveratrol treatments	330 mg/day, 3 days	Coronary artery bypass graft patients had higher FMD than those who had undergone percutaneous coronary intervention, whereas percutaneous coronary intervention patients showed no difference in FMD	[115]
Hypertension	Patients with hypertension (*n* = 24) given a placebo or resveratrol	300 mg, acute supplementation	Increased FMD in women and individuals with higher LDL-C	[116]
	Patients with hypertension (*n* = 18) given a placebo or isolated phytochemicals	60 mg/day, 28 days	Decreased diastolic blood pressure	[117]
	Peripheral artery disease patients were split into two groups and given either a standard balloon angioplasty (*n* = 75) or a resveratrol drug-coated balloon (*n* = 78)	0.9 µg/mm^2^, 728 days	Target lesion revascularization was reduced, and patients were able to walk further after treatment than those who received standard balloon angioplasty	[118]
Diabetes	A prospective, open-label, randomized controlled experiment involving 62 patients with type 2 diabetes	250 mg/day, 90 days	Decreases in hemoglobin A1c, systolic blood pressure, total cholesterol, and total protein indicate better glycemic control	[119]
	Placebo-treated (*n* = 38) and resveratrol-treated (*n* = 38) patients with type 2 diabetes	1000 mg/day, 56 days	Changes in plasma HDL-C, TG, TC, and LDL-C were not significant, whereas plasma glucose was reduced	[120]
	A randomized, placebo-controlled, double-blind investigation of 19 patients with type 2 diabetes	5 mg twice daily, 30 days	Glucose and insulin levels dropped, glucose spikes after meals were postponed, and ortho-tyrosine was excreted in the urine	[121]
	Nonalcoholic fatty liver disease patients who were overweight and randomly assigned to either a placebo (*n* = 8) or resveratrol (*n* = 8) group	1500 mg/day, 180 days	Very low-density lipoprotein TG secretion. Oxidation, and clearance rates were not affected, neither at baseline nor in response to insulin	[122]
	Type 2 diabetes patients in whom the disease is under control (*n* = 17) were given either placebo or resveratrol	150 mg/day, 30 days	Insulin sensitivity in the liver and the rest of the body did not change, nor did the amount of fat stored in the liver	[123]
	Placebo- and resveratrol-treated patients with type 2 diabetes (*n* = 14)	1000 mg/day, 35 days	Glycemic control and glucagon-like peptide 1 secretion did not vary	[124]
	Treatment with resveratrol or a placebo in elderly people with glucose intolerance (*n* = 30)	2–3 g/day, 42 days	Reactive hyperemia index rises, but blood pressure and plasma lipid levels remain unchanged	[125]
	Diabetic patients at high risk (*n* = 8) treated with placebo and resveratrol	150 mg/day, 34 days	There was no difference in the absorption of 18F-fluorodeoxyglucose or the inflammation of arteries	[126]
Obesity	Children and adolescents with obesity were split into two groups: those who took a resveratrol supplement (*n* = 16) and those who took a placebo (*n* = 11)	20 mg/day, 180 days	Enhanced hyperemic delta flow 6 months after post-occlusive release	[127]
	Obese older people (*n* = 22) were divided into two groups: those given placebo or resveratrol with curcumin	200 mg, 30 min before consuming the high-fat meal	Post-meal soluble vascular cell adhesion molecule-1 response was reduced, but other inflammatory indicators and adhesion molecules in the blood were unaffected	[128]
	Placebo (*n* = 10), 300 mg (*n* = 10), and 1000 mg (*n* = 9) resveratrol groups were used to test the effects of resveratrol on the weight and health of older, overweight persons	300 and 1000 mg/day, 90 days	The 1000 mg resveratrol group had higher levels of soluble vascular cell adhesion molecule-1 and total plasminogen activator inhibitor than the 300 mg resveratrol and placebo groups	[129]
Neurodegenerative diseases	102 people with early-onset Huntington’s disease (HD)	40 mg twice a day, 365 days	Not known yet	[130]
	120 patients with mild to moderate dementia most likely due to Alzheimer’s disease (AD)	500 mg/day with dose escalation of up to 1000 mg twice/day, 365 days	Nausea, weight loss, and diarrhea are the only reported side effects of resveratrol, which is safe and well-tolerated. CSF Aβ40 and Aβ42 biomarkers show no improvement. Enhanced decline in brain volume	[131]
	27 people with mild to moderate AD	Resveratrol, glucose, and malate supp. delivered in grape juice, 365 days	At modest doses, resveratrol is safe and well-tolerated. The Mini-Mental State Exam and the AD Assessment Scale for Cognition scores did not significantly change	[130]
Cancer	14 patients with prostate cancer	500, 1000, 2000, 3000, or 4000 mg of MPX. Every 500 mg MPX has 4.4 μg resveratrol, 60–930 days (depending on the patient)	Increased PSADT	[132]
	A single-center, randomized, placebo-controlled trial of 66 people with prostate cancer	150 mg or 1000 mg daily, 120 days	A drop in androstenedione, and dehydroepiandrosterone (DHEAS). The PSA and prostate size remained unchanged	[133]
	Phase 1 trial of nine patients with colorectal cancer; randomized, placebo-controlled, double-blind	5.0 g SRT501, 14 days before surgery	Elevated levels of activated caspase-3 (apoptosis)	[134]
	Cases of colorectal cancer in 20 patients	500 or 1000 mg, 8 days prior to surgery	Ki-67 staining decreases, indicating a decrease in tumor cell growth	[135]
	Randomized, double-blind, placebo-controlled clinical study for breast cancer in 39 people	5 or 50 mg twice daily, 90 days	Decreased *RASSF-1α* methylation	[136]

TG: triglyceride; TC: total cholesterol; HDL-C: high-density lipoprotein cholesterol; ox-LDL: oxidized low-density lipoprotein; LDL-C: low-density lipoprotein cholesterol; FMD: flow-mediated dilatation; PSA: prostate-specific antigen; MPX: pulverized muscadine grape skin, which contains resveratrol; PSA: doubling time.

**Table 3:** Recent nanoformulation-based advances in boosting resveratrol’s biological activity.

Nanoformulation Method	Study Model	Outcome	Reference
Resveratrol medication delivery systems based on self-emulsification	In vitro, in vivo (rats)	Enhanced pharmacokinetics, decreased metabolism, and enhanced solubility	[161]
Micellar solubilization of resveratrol	Twelve healthy volunteers (oral administration)	Increased oral bioavailability	[162]
Suspension of free resveratrol, resveratrol-filled nanoparticles, and layer-by-layer nanoparticles	In vivo (Wistar rats, oral administration, 20 mg/kg)	Systemic exposure was increased when resveratrol was encapsulated in layer-by-layer nanoparticles with resveratrol nanocores	[163]
Nanoparticle delivery method based on oat-shellac proteins	In vitro, in vivo (rat model)	Resveratrol was buffered in the stomach acid and released gradually into the small intestine. Transport and absorption by cells are enhanced relative to free resveratrol. Enhancement in bioavailability	[164]
Resveratrol nanoencapsulation in casein	In vitro, in vivo (rats)	Oral administration in rats: remained in the gut and reached intestinal epithelium. Produced high plasma levels of resveratrol (sustained for at least 8 h) and similar results for its metabolites. Oral bioavailability was 10 times higher compared to an oral solution of resveratrol	[165]
*Trans*-resveratrol nanocrystals	In vitro, in vivo (rats)	Increased oral bioavailability	[166]
Nanoparticles of human serum albumin coupled with glycyrrhizic acid and loaded with resveratrol	In vivo (rats; single-dose tail vein injection)	The absorption rate of resveratrol was increased. High levels of resveratrol were found in most of the rats’ vital organs. The highest levels were found in the liver, suggesting that a delivery method that focuses on the liver could be effective	[167]
*Trans*-resveratrol-loaded mixed micelles	In vivo (rats; intravenous administration)	Enhanced pharmacokinetic parameters. Improved brain targeting	[168]
Resveratrol bovine serum albumin nanoparticles (RES-BSANP)	In vivo (nude mice; intraperitoneal injection)	Enhanced dilution and soluble in water. Cancer development was suppressed in hairless mice bearing human ovarian primary tumors	[169]
Folate-conjugated HSA nanoparticles	HePG2 liver cancer cells	Showed decreased resveratrol release and increased cytotoxicity	[170]
Piperine-loaded mixed micelles	MCF-7 breast cancer cells	Improved cytotoxicity	[171]
Sericin nanoparticles	Caco-2 cells colorectal cancer cells	Strong cytotoxic against Caco-2 cells	[172]
Folic acid-targeted micelles	MCF-7 breast cancer cells	Increased cytotoxicity was achieved due to the sustained release of encapsulated resveratrol provided by the nano-formulation.	[173]

## Data Availability

Not applicable.
